# 2021: the year [^177^Lu]Lu-PSMA-617 RLT PSMA is ready for incorporation into clinical guidelines? Reply to “A perspective on the EANM procedure guidelines for radionuclide therapy with 177Lu-labelled PSMA-ligands” by Dr. Germo Gericke

**DOI:** 10.1007/s00259-021-05409-w

**Published:** 2021-05-22

**Authors:** Ken Herrmann, Clemens Kratochwil, Wolfgang P. Fendler, Matthias Eiber, Roland Hustinx

**Affiliations:** 1 Department of Nuclear Medicine, Universitätsklinikum Essen, Essen, Germany; 2 German Cancer Research Center and Department of Nuclear Medicine, Universitätsklinikum Heidelberg, Heidelberg, Germany; 3 Klinikum rechts der Isar, Technische Universität München, Munich, Germany; 4 EANM Board & Division of Nuclear Medicine, University Hospital of Liège, Liège, Belgium

Dear Sir,

Thank you very much for giving us the opportunity to comment on the important aspects raised by Dr. Gericke.

The intention of the “EANM procedure guidelines for radionuclide therapy with ^177^Lu-labelled PSMA-ligands (^177^Lu-PSMA RLT)” is to give guidance to centers intending to implement PSMA-targeted radioligand therapy prior to availability of an approved compound [1]. While not clearly defined the labelling as “procedure guideline” aims to describe the process of patient selection, application and follow-up. It is not intended to discuss or to draw definitive conclusions on efficacy comparing different therapies, which is in the scope of randomized clinical trials. The preamble of procedure guidelines clearly defines a flexible framework and significant impact of advances in knowledge or technology subsequent to publication of the guidelines on clinical practice. The recently published TheraP data as well as the press release by Novartis raise the expectations that soon prospective phase 3 data will be available justifying the inclusion of [^177^Lu]Lu-PSMA-617 RLT into clinical (as compared to procedure) guidelines [2, 3].

The mentioned statement “exchangeable application of PSMA-617 and PSMA-I&T” clearly relates to the mode of application, the expected toxicity profile and the known dosimetry data. We fully agree with Dr. Gericke that there is no comparative data available, nor does the limited body of available data allow for a reliable clinical comparison of biodistribution.

Dr. Gericke also questions whether the statement “data indicate a favourable safety profile for ^177^Lu-PSMA RLT” can be generalized across various ligand molecules. Despite the tremendously higher number of patients historically treated with [^177^Lu]Lu-PSMA-617, there are nevertheless more than 100 patients treated with [^177^Lu]Lu-PSMA-I&T reported in the literature [4].

Regarding the comment of Dr. Gericke on the “efficacy” part, we concur that with the recently published data by Hofman et al. as well as the soon expected VISION trial data, [^177^Lu]Lu-PSMA-617 is the only compound that has, for now, clinical supportive evidence for efficacy [2, 3]. Accordingly, the integration of [^177^Lu]Lu-PSMA-617 into clinical guidelines can be expected.

The authors want to reemphasize that a procedural guideline is intended to assist Nuclear Medicine practitioners in performing, interpreting and reporting procedures which are either not yet or — from a procedural standpoint — not adequately represented in clinical guidelines. Procedural guidelines do not intend to give recommendations regarding clinical efficacy or clinical superiority. To adequately clarify this, an EANM wide definition and standardization of the various types of guidelines are under its way.

Moreover, the authors fully support and welcome the activity of AAA/Novartis in the field of radioligand therapy and fully echo the commitment to adhere to scientific standards, precise interpretation of available information, or lack thereof, and timely updates as new information becomes available. The unique space of radioligand therapy strongly benefits from strong academia efforts in developing novel concepts potentially accelerating the access of cancer patients to new effective therapies. However, this space also needs the expertise, credibility and track record of newly and well-established pharmaceutical companies to allow for worldwide approval and access to radioligand therapy. The current growth of radioligand therapy will only be sustainable if the investments made will be economically rewarded — for Nuclear Medicine, this means if an approved drug is available, it has to be used clinically.

In summary, we hope we have successfully clarified the intent and scope of the ^177^Lu-PSMA RLT procedure guideline [1]. In anticipation of the presentation of the VISION results, the authors sincerely are convinced that the use of [^177^Lu]Lu-PSMA-617 RLT will soon find its way into clinical guidelines.

## Data Availability

Not applicable.
